# The Incidence and Differential Seasonal Patterns of *Plasmodium vivax* Primary Infections and Relapses in a Cohort of Children in Papua New Guinea

**DOI:** 10.1371/journal.pntd.0004582

**Published:** 2016-05-04

**Authors:** Amanda Ross, Cristian Koepfli, Sonja Schoepflin, Lincoln Timinao, Peter Siba, Thomas Smith, Ivo Mueller, Ingrid Felger, Marcel Tanner

**Affiliations:** 1 Swiss Tropical and Public Health Institute, Basel, Switzerland; 2 University of Basel, Basel, Switzerland; 3 Population Health and Immunity Division, Walter and Eliza Hall Institute, Parkville, Australia; 4 Papua New Guinea Institute of Medical Research, Goroka, Papua New Guinea; 5 ISGlobal, Barcelona Centre for International Health Research (CRESIB), Barcelona, Spain; 6 Department of Medical Biology, University of Melbourne, Melbourne, Victoria, Australia; 7 Department of Parasites and Insect Vectors, Institut Pasteur, Paris, France; Johns Hopkins Bloomberg School of Public Health, UNITED STATES

## Abstract

*Plasmodium vivax* has the ability to relapse from dormant parasites in the liver weeks or months after inoculation, causing further blood-stage infection and potential onward transmission. Estimates of the force of blood-stage infections arising from primary infections and relapses are important for designing intervention strategies. However, in endemic settings their relative contributions are unclear. Infections are frequently asymptomatic, many individuals harbor multiple infections, and while high-resolution genotyping of blood samples enables individual infections to be distinguished, primary infections and relapses cannot be identified. We develop a model and fit it to longitudinal genotyping data from children in Papua New Guinea to estimate the incidence and seasonality of *P vivax* primary infection and relapse. The children, aged one to three years at enrolment, were followed up over 16 months with routine surveys every two months. Blood samples were taken at the routine visits and at other times if the child was ill. Samples positive by microscopy or a molecular method for species detection were genotyped using high-resolution capillary electrophoresis for *P vivax MS16* and *msp1*F3, and *P falciparum msp2*. The data were summarized as longitudinal patterns of success or failure to detect a genotype at each routine time-point (eg 001000001). We assume that the seasonality of *P vivax primary* infection is similar to that of *P falciparum* since they are transmitted by the same vectors and, because *P falciparum* does not have the ability to relapse, the seasonality can be estimated. Relapses occurring during the study period can be a consequence of infections occurring prior to the study: we assume that the seasonal pattern of primary infections repeats over time. We incorporate information from parasitological and entomology studies to gain leverage for estimating the parameters, and take imperfect detection into account. We estimate the force of *P vivax* primary infections to be 11.5 (10.5, 12.3) for a three-year old child per year and the mean number of relapses per infection to be 4.3 (4.0, 4.6) over 16 months. The peak incidence of relapses occurred in the two month interval following the peak interval for primary infections: the contribution to the force of blood-stage infection from relapses is between 71% and 90% depending on the season. Our estimates contribute to knowledge of the *P vivax* epidemiology and have implications for the timing of intervention strategies targeting different stages of the life cycle.

## Author Summary

*Plasmodium vivax* is the most widespread of the malaria species affecting humans. It has the ability for parasites to lie dormant in liver cells and then to relapse weeks or months later, causing further blood-stage infections and onward transmission. Relapses present a challenge to control and elimination programs. The contribution of relapses to the force of blood-stage infection is not well established. While genotyping can distinguish individual infections, the difficulty lies in the inability to distinguish primary infections (occurring shortly after an infectious mosquito bite) and relapses. This is a gap in the knowledge of the epidemiology of *P vivax*. We develop a statistical model to tease out and estimate the contributions of primary infections and relapses to the force of blood-stage infection. We use data from a cohort of children in Papua New Guinea with genotyped routine blood samples. The study area has both *P vivax* and *P falciparum* malaria: we use the seasonality of *P falciparum* to estimate the seasonality of *P vivax* primary infections. We also take into account infections occurring prior to the study period and their subsequent relapses during the study period. We find that approximately 80% of the force of blood-stage infection l is contributed by relapses and that primary infections and relapses have different seasonal patterns. The findings are important to the epidemiology of *P vivax* and for designing intervention strategies targeting different stages of the parasite life cycle.

## Introduction

Globally, over a billion inhabitants are exposed to *Plasmodium vivax* and suffer from an estimated 70 to 80 million cases per year [1]. It has been increasingly recognized that *P vivax* can cause severe and fatal disease [2,3]. Effective control measures are needed. Designing intervention strategies requires estimates of the incidence of blood-stage infection from both primary infection and relapses resulting from the ability of *P vivax* to remain dormant in liver cells for weeks or months. However, these processes, and the longitudinal patterns they give rise to, have not been well quantified in natural populations.

The timing of relapses varies by geographic region although the triggers have not been established [4]. Information on the patterns of relapse have come from studies of the deliberate infection of non-immune adult neurosyphilis patients and healthy volunteers, clinical episodes in travellers and soldiers returning from endemic areas and primaquine studies [4,5]. Most of these studies assume that in individuals with little immunity, relapses will cause symptoms, but this has not been established. Few data are available to date on infection in children and adults living in endemic areas who are likely to have acquired some immunity and may harbour multiple *P vivax* infections.

Cohort studies in natural populations provide a source of information on *P vivax* infection dynamics. The infecting genotypes can be distinguished on the basis of highly polymorphic genetic markers. However, the analysis of such data is not straightforward because the detection of genotypes is imperfect, even when using polymerase chain reaction (PCR). Several methods have been developed to estimate *P falciparum* infection, clearance, duration of infections and detectability [6–11]. *P vivax* presents additional challenges due to the relapses, which cannot be distinguished from the primary infections by genotyping [12].

We present estimates of the relative contributions of primary infection and relapses to the force of blood-stage infection in a cohort of children in Papua New Guinea aged one to five years [13], for the first time estimating these quantities in a cohort without primaquine treatment. The analyses indicate how these relative contributions vary seasonally, allowing for age, detectability, treatment and insecticide-treated net (ITN) use. Previous studies have summarized the observed parasitology and clinical incidence [13], the force of blood-stage infection [14], the detectability [15], and described the multiplicity of infection [16] in this cohort. To disentangle the contributions of primary infection and relapse, we use this information and gain additional leverage for estimating the parameters from complementary entomology studies and experimental and therapeutic infections.

## Methods

### Study design, sample collection and genotyping

The cohort study was carried out in Ilaita, Maprik district, Papua New Guinea. Two hundred and sixty-four children aged between one and three years at enrolment were followed up over 16 months [13]. The prevalence by light microscopy at enrolment was 44% for *P vivax* and 33% for *P falciparum*, with an estimated 2.46 *P vivax* and 2.56 *P falciparum* episodes per child per year [13].

Finger prick blood samples were collected for genotyping at nine routine survey time-points at two-month intervals. The first and last surveys comprised single blood samples whereas at the second to eighth routine time-points, two samples were taken 24 hours apart. Additionally, blood samples were taken if the child was ill, either when presenting for treatment or during fortnightly active case detection visits. Coartem was provided by the study team to those who were RDT positive and had fever or history of fever in the last 48 hours. Further antimalarial drugs were provided outside the study and were recorded in the child’s health book.

DNA was extracted as described previously [15]. High-resolution genotyping by PCR followed by capillary electrophoresis was carried out for one *P falciparum* marker, merozoite surface protein 2 (*msp2*), and two *P vivax* markers (*msp1*F3 and MS16). The methods have been described elsewhere [16,17]. Only blood samples positive for *P falciparum* or *P vivax* by microscopy or by post-PCR ligase detection reaction (LDR), a molecular method for Plasmodium species detection [18], were genotyped. This was justified by the very low proportion of negative samples which were positive by genotyping [16].

### Ethics statement

The cohort study was approved by institutional review boards of the PNG Medical Research Advisory Committee (approvals 05.19 and 09.24), University Hospitals Case Medical Center (Cleveland, Ohio USA), and the Ethikkommission beider Basel (approval 03/06). Informed written consent was provided by the parents or legal guardians of each child.

### Data preparation

**Fig 1:**
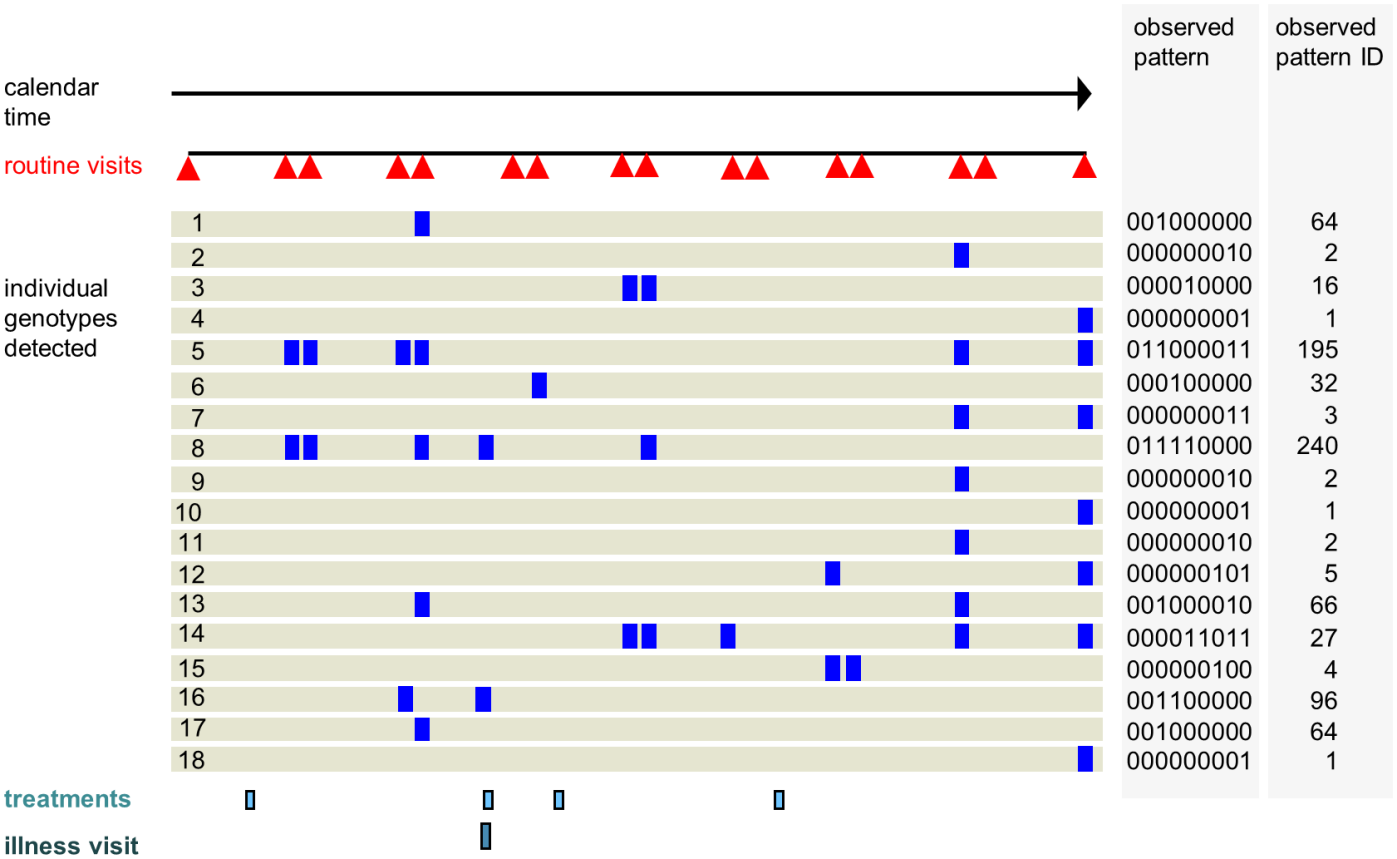
Example of the structure of the data. The diagram represents the data for a child who had no missing visits. The time of the routine visits is shown by red triangles: the timepoints were spaced two months apart with two visits 24 hours apart for all but the first and last timepoints. The child had had a sample taken at each routine visit. Eighteen distinct genotypes were detected, each is shown with blue rectangles representing the visits where the genotypes where detected. The timing of an illness visit (light blue rectangle) and recorded treatments (dark blue rectangle) is shown below. The column for observed patterns shows how the data are represented for the analysis: 0 indicates that the genotype was not detected at that routine time-point and 1 that it was detected. The pairs of routine visits 24 hours apart were combined, and the detectability was taken into account. The observed pattern ID is the distinct number assigned to the specific pattern.

For the observed data, we denote an observed genotype as 1 and an undetected or absent genotype as 0. For the 9 time-points, there are 512 possible patterns which were numbered from 0 to 511 using their binary value (eg 000000100 is pattern 4). This yielded a frequency distribution of binary patterns for each child, to which statistical models could be fitted (Fig 1). We restricted the analysis to participants who provided a blood sample on at least one day at each of six or more routine time-points. Where treatment was given following the first visit of a routine 24 hour pair, the second visit was excluded.

Blood samples were also collected and genotyped if a child was ill at non-routine visits. In the analysis, we assigned genotypes from visits prompted by illness to the next routine time-point (we define month intervals to start just after the routine time-point). Correspondingly, we include the treatment but timed to occur just after the routine time-point. This is equivalent to assuming that the blood-stage infection would have persisted up to this time-point in the absence of treatment. This may cause some detection bias since clinical symptoms for malaria are more likely in those with less acquired immunity and because seeking treatment for both malaria and other illnesses is associated with distance to the health facility. However excluding these genotypes would also cause bias since, in most cases, they were treated and the genotypes would not be present at the next routine time-point. The bias may increase the estimated incidences.

The model does not allow for reinfection of a child with the same genotype. For the analysis of MS16, this was justified by the low frequencies of most alleles, with the most common allele at baseline in this cohort having a frequency of only 5% compared with 24% for the most frequent allele of *P vivax* msp1F3 and 15% for *P falciparum msp2* [16,19]. For this reason the primary analysis (including estimates of the force of infection) of *P vivax* focuses on the marker MS16. For *P vivax msp1F3* we report the sensitivity of the estimates to the exclusion of the four most common alleles We omit alleles with frequencies greater than 5% from analyses of the seasonality of *P falciparum msp2*.

### The seasonality of *P vivax* primary infections is estimated from the seasonality of *P falciparum* infections

We assume that the seasonal pattern of *P falciparum* infections provides approximate estimates of the seasonal pattern of *P vivax* primary infections since (i) *P vivax* and *P falciparum* are transmitted by the same vectors and (ii) we assume that seasonality is driven primarily by vector densities and that parasites in the human host are less important. The validity of this assumption was investigated using entomological and parasitological data from the Wosera, Papua New Guinea (S1 Text).

Thus models for both *P vivax* and *P falciparum* are required. We describe the general structure of these models followed by their specific features.

### General structure of the models for infection dynamics

We use Markov models to estimate the infection dynamics. The processes determining the modelled frequencies of the binary patterns of the genotypes are acquisition (via primary infection or relapse), clearance of the blood-stage infections and detection.

Following [6,8,9], each genotype, occurring in each child, can be present or absent at each of the 9 routine study time-points leading to 2^9^(512) possible true patterns. The true patterns are denoted by *t*_*cs*_ for child *c* and pattern *s* = 0,…,511. The probability of a particular true pattern, Pr(*t*_*cs*_) may vary between children due to covariates such as treatment, age and ITN use.

The state of the genotype at each time-point *i*, whether 1 or 0, is denoted by *d*_*csi*_. The probability of each true pattern, Pr(*t*_*cs*_), is the product of the probability of the states at each time-point, $\text{Pr}(t_{cs})\;=\;\prod_{i}\text{Pr}\left(d_{csi}\right).$ The probability of the initial state is estimated as a parameter and the transition probabilities for each two month period are derived from the parameters for clearance and infection for the specific model (described in later sections) using equations from Munch [20].

Once the probabilities of the true patterns have been calculated, we then take detectability into account to obtain the probability of each observed pattern *o*_*cw*_ for child *c* and pattern *w* = 0,…511. The probability of detecting a genotype given that it is present at time-point *i* in child *c*, *z*_*ci*_, depends on the probability of detecting the genotype in a single blood sample, (*q*_*g*_ for samples which were genotyped because they were microscopy or LDR positive and *q*_*k*_ for samples which were negative and not genotyped), and the corresponding numbers of blood samples *g*_*ci*_ and *k*_*ci*_,

$$z_{ci}=1-\left({\left(1-q_{g}\right)}^{g_{ci}}\;{\left(1-q_{k}\right)}^{k_{ci}}\right).$$

We fix the value of *q*_*g*_ to previous estimates from this cohort obtained from an analysis of the samples taken 24 hours apart [15]: 0.79 (0.76, 0.82) for *P falciparum msp2*, 0.61 (0.58, 0.63) for *P vivax* MS16 and 0.73 (0.71, 0.75) for *P vivax msp1*F3. Where two samples 24 hours apart were both genotyped and combined for one routine timepoint, the probability of detecting a genotype in at least one sample is estimated to be 1−(1−*q*_*g*_)^2^, a special case of the equation above. Since there was a very low proportion of samples negative by microscopy and LDR which were found to be positive [16], we set *q*_*k*_ to 0.99. Detectability has been found to vary by age in cohorts spanning all ages [6, 9], however the variation is expected to be small over the narrow age range in this cohort. We acknowledge that detectability may decrease with acquired immunity and vary between primary infections and relapses but we assume a constant for these analyses.

The probability of observing pattern *o*_*cw*_ conditional on the true pattern *t*_*cs*_, Pr(*o*_*cw*_ | *t*_*cs*_), is obtained by multiplying the probability of the true pattern by the probability of detection,

$$\text{Pr}(o_{cw}|\;t_{cs})\;=\text{ Pr}(t_{cs})\;\prod_{i}^{}f\;(b_{cwi},d_{csi})$$

where *b*_*cwi*_ is the observed state at time-point *i*, and

$$f(b_{cwi},d_{csi})\;=\{\begin{array}{lllll} 1 & if\;b_{cwi} & =0 & \text{and }d_{csi} & =0\\ 0 & if\;b_{cwi} & =1 & \text{and }d_{csi} & =0\\ 1-z_{ci} & if\;b_{cwi} & =0 & \text{and }d_{csi} & =1\\ z_{ci} & if\;b_{cwi} & =1\; & \text{and }d_{csi} & =1 \end{array}$$

The probability of observed pattern, Pr(*o*_*cw*_), is given by summing over the conditional probabilities given each of the possible true patterns,

$$\text{Pr}(o_{cw})\;=\;\sum_{s=0}^{511}\text{Pr}(o_{cw}\;|\;t_{cs})\text{ Pr}(t_{cs})\;s=0,\ldots .511$$

We assume a multinomial distribution for the frequency of the observed patterns *n*_*c*0_, *n*_*c*1_, …..*n*_*c*511_ ~ *Mn*(*N*, Pr(*o*_*c*0_), Pr(*o*_*c*1_), … … Pr(*o*_*c*511_)) where *N* is the number of possible genotypes per child, which is taken from the number of observed genotypes for all children in the cohort.

The log likelihood (LL) is given by

$$LL\;=\;\overset{C}{\underset{c=1}{\sum }}\overset{511}{\underset{w=0}{\sum }}Pr\left(o_{cw}\right)n_{cw}$$

where *n*_*cw*_ is the frequency of pattern *w* for child *c* and *C* is the number of children in the study. Missing time-points were included since the number of visits at the time-point, and therefore the detectability, was set to zero.

### Estimating the seasonality of the incidence of *P falciparum* infections

Following earlier approaches [6,8,9], we assume that the observed *P falciparum* patterns must be derived from a restricted set of true patterns which assume that negative routine time-points bracketed by positive time-points are attributable to a lack of detection (this is not true for *P vivax* due to relapses). Only the 143 children with at least one visit at each study time-point are included in the *P falciparum* seasonality analysis. We allow the incidence of *P falciparum* infections to vary seasonally: for each interval leading up to time-point *i*, the incidence of infection with a *P falciparum* genotype, λ_*fci*_, was estimated.

The probabilities were derived for transitions from 0 to 1, and 1 to 0, via equations from Muench [20],

$$p_{fci\;}^{01}\;=\;\frac{\lambda_{fci}}{\lambda_{fci}+\;\mu_{f}}\;(1-e^{-(\lambda_{fci}\;+\;\mu_{f})m_{ci}})$$

$$p_{fci\;}^{01}\;=\;\frac{\mu_{f}}{\lambda_{fci}+\;\mu_{f}}\;(1-e^{-(\lambda_{fci}\;+\;\mu_{f})m_{ci}})$$

where *μ*_*f*_ is the clearance rate for *P falciparum* infections, λ_*fci*_ is the mean infection rate for a *P falciparum* genotype for child *c* in interval *i* and *m*_*ci*_ is the duration at risk in two-month units. The probabilities $p_{fci}^{00}$ and $p_{fci}^{11}$ are given by ${1-p}_{fci}^{01}$ and $1-p_{fci}^{10}\;$ respectively.

Treatment was found to affect the seasonal pattern slightly and was included in the model. We also adjusted for different biting rates by age using body surface area.

### Model for *P vivax* infection dynamics

Acquisition of *P*. *vivax* blood-stage infection can be either by primary infection or relapse. Since we assume that reinfection with the same genotype is negligible (as frequent alleles were excluded in the case of *msp1*F3), it follows that the earliest blood-stage infection from a genotype could be due to a primary infection or relapse and that subsequent infections of the same genotype must be due to relapses. The primary infection of a genotype relapsing during the study period occurred either during the study period or before follow-up began. We take primary infections occurring prior to the study into account by extending the numbers of time-points to include eight notional pre-study two-monthly time-points, spanning the 16 months preceding enrolment making a total number of notional pre-study and actual routine time-points of17. We chose a period of 16 months for the pre-study period so that sufficient time was allowed for the modelled primary infections to minimally constrain the subsequent relapses occurring during the study period. The pre-study period timepoints are set two months apart since they must capture the seasonality of the primary infections (which was estimated at two month intervals, the spacing of timepoints during the study period). For a pre-study time-point before the child was born, the probability of a genotype being present is set to zero.

The probability of a true *P vivax* genotype pattern is given by the probability that the primary infection occurred in interval *y*, *p*_*yc*_, for *y* = 0, …16, multiplied by the probabilities of the subsequent transitions driven by clearance of blood-stage infections and relapses. We assume that the primary infection always occurs soon after the inoculation, but may not necessarily be detected or occur at the time of the routine time-point.

$$Pr\left(t_{cs}\right)\;=\;\prod_{i}Pr\left(d_{csi}\right)\;=\;p_{yc}\prod_{i>y}Pr\left(d_{csi}\right)$$

We estimate the seasonal pattern and magnitude of *p*_*yc*_.

### The incidence of *P vivax* primary infections

The incidence of *P vivax* primary infection with a genotype in interval *i* is λ_*vi*_ = *β*_1_ λ_*fi*_, where *β*_1_ represents a constant to calculate the number of *P vivax* primary infections based on the seasonality of *P falciparum* infection. *β*_1_ is estimated. The seasonality was estimated for the observed study period. It was assumed to repeat with an annual cycle and was extended backwards to the eight notional two-month intervals prior to the study period for the *P vivax* model.

If the genotype has not been present up until interval *i*, then the probability of a primary infection during interval *i* is $1-\;e^{\lambda_{vi}\;m_{ci}}$ and the probability that a primary infection does not occur is $e^{\lambda_{vi}\;m_{ci}}$. Thus the probability that a primary infection occurs in interval *y* is $p_{yc}\;=\;(1-\;e^{-\lambda_{vyc}m_{ci}})\;\prod_{i\;=\;1}^{y-1}e^{-\lambda_{vyc}m_{ci}}$. We do not adjust *p*_*yc*_ for treatment.

### The clearance of *P vivax* blood-stage infections

We used a value for *μ*_*b*_, the rate of clearance of untreated blood-stage infections, of 0.8 per two month interval, corresponding to a mean duration of blood-stage infection of 76 days estimated from malaria therapy patients (Ross et al, in prep). This value is consistent with an estimate from a different cohort in Papua New Guinea [21]. *μ*_*b*_ could not be reliably estimated from the cohort data due to very frequent treatment of blood-stage infections. In the model, we assume that a relapse does not affect the duration of an ongoing blood-stage infection with the same genotype, and that the mean durations of blood-stage infections resulting from relapses and primary infections are the same. Similarly, *μ*_*b*_, does not change with age or acquired immunity, based on findings in *P falciparum* [22].

Treatment was assumed to terminate the blood-stage infection. The child was assumed to be at risk of a new blood-stage infection from 14 days after the last treatment to allow for the prophylactic period. If there was an illness visit with *P vivax* genotypes detected and the genotypes were combined with the following routine timepoint, then the treatment was assumed to occur just after the subsequent routine timepoint.

### The incidence of relapse by time since primary infection

We allow the incidence of relapse to vary by time after the primary infection. We treat relapses as independent both between genotypes and within a genotype. The incidence of relapse is also independent of the presence of blood-stage infection with the same genotype. Early relapses may occur in the same two-month interval as the primary infection [23–25]. Primary infections are modelled as occurring half way through the interval.

*γ*_*yci*_ is the incidence of relapses in interval *i* conditional on the primary infection having occurred in interval *y* for *i*−*y* = 1, …7. The transition probabilities are again derived from the rates using the equations from Muench [20],

$$p_{yci\;}^{01}\;=\;\frac{\gamma_{iyc}}{\gamma_{iyc}+\;\mu_{b}}\;(1-e^{-(\gamma_{yci}\;+\;\mu_{b})m_{ci}})$$

$$p_{yci\;}^{10}\;=\;\frac{\mu_{b}}{\gamma_{yci}+\;\mu_{b}}\;(1-e^{-(\gamma_{yci}\;+\;\mu_{b})m_{ci}})$$

We initially tried to estimate the distribution of relapse times from the Papua New Guinea cohort. However we were unable to robustly estimate the incidence of relapse in the interval in which the primary infection occurs, probably since primary infections have a higher probability of leading to clinical illness, and therefore being picked up during illness visits, and we include genotypes from illness visits at the subsequent routine time-point with treatment afterwards. We were also unable to robustly estimate the incidence of relapses between 12 and 16 months after the primary infection, probably due to insufficient data. We therefore constrained the time-pattern of the incidence of relapses using data from Coatney *et al* [26] on the distribution of the incidence of relapse, *f*(*x*) (S2 Text). This distribution was consistent with the pattern of our preliminary findings for months two to twelve. We estimate a parameter,*β*_2_, which acts as a scale factor to obtain the absolute incidence of relapse so that *γ*_*iyc*_ = *f*(*i*−*y*) *β*_2_ in the absence of covariates. A list of quantities in the model is given in S1 Table.

### Covariates

We considered a limited number of covariates in the *P vivax* model (Table 1).

**Table 1:** Covariates included in the *P vivax* model and their action.

Covariate	Effect(s)
Age	The rate of primary infection is scaled by body surface area, estimated using WHO growth standards for weight and height[27] and the Mosteller formula [28]. Age is additionally a potential covariate for the incidence of relapse (</≥36months). We assume no maternal immunity on liver-stage parasites, nor prenatal infections.
Treatment	Effective treatment is assumed to terminate all blood stage infections. The probability of a genotype causing a new blood-stage infections at the subsequent routine time-point (a transition of 0 to 1) is calculated using the time at risk from the end of the prophylactic period to the routine timepoint Treatment is assumed not to affect the pre-erythrocytic stages. If there was an illness visit with *P vivax* genotypes detected and the genotypes were combined with the following routine timepoint, then the treatment was assumed to occur just after the subsequent routine timepoint
ITN use	Ratio for the incidence of primary infection. ITN use during the study period also applies to the pre-study period^1^
Village	Ratio for the incidence of primary infection in Sunuhu compared to Ilaita (regions within the study area)

^1^There was no special ITN campaign in the pre-study or study periods

Different assumptions about the effect of antimalarial treatment were possible. Coartem was provided by the study team to those who were RDT positive and had fever and history of fever. Treatment was also sought in the health centres and recorded in the health book. We assumed that monotherapy with amodiaquine, chloroquine, SP and cotrimoxazole had negligible effects and did not include the small number of these as treatments. The majority of treatments included were coartem or artesunate combinations (80%) and amodiaquine and sulphadoxine-pyrimethamine (AQ-SP, 20%). We assumed that these treatments resulted in a parasitological cure. Where treatment was given following the first visit of a 24 hour pair, the second visit was excluded. Small changes in the assumptions about treatment did not alter the conclusions, however increasing the assumed prophylactic period did increase the estimated incidence of relapse and to a lesser extent the estimated incidence of primary infection.

We also attempted to adjust the *P vivax* incidence of relapse for the presence of a *P falciparum* fever, any malaria fever, or any fever, in the previous interval.

### Implementation

We maximised the exact log likelihood using the Nelder-Mead algorithm [29,30] comparing the observed frequencies of patterns with the expected frequencies summed over all genotypes and all children. Confidence intervals were calculated using profile likelihood [31,32]. The program was written in C++.

## Results

### Descriptive summary of the Ilaita I cohort data

A total of 264 children were enrolled into the cohort, 189 at the time of the first survey. Routine survey follow-up was high: on average 95% of those enrolled were present at each of the routine visits[13]. Of the 264 enrolled, 243 (92%) were seen at least once at six or more routine study time-points and 143 (54%) children were seen at least once at all nine routine time-points. The children seen at least once at 6 or more routine time-points contributed a total of 2003 routine time-points, 656 (33%) with one visit and 1347 (67%) with two visits 24 hours apart. There were 1038 intervals which included visits made due to illness. Treatment was given during a median of 4 intervals per child (range = 0,9).

There were 114 *P vivax* MS16 alleles detected, the most common had a frequency of 5% in the baseline survey. There were 63 *msp1*F3 alleles, with the highest frequency of 24% and 55 *P falciparum msp2* with the highest frequency of 15% (Table 2). Omitting alleles which were more frequent than 5% left us with 59 *msp1*F3 and 50 *msp2* alleles. The lower numbers of msp1F3 compared to MS16 genotypes (Table 2) suggests that the former locus is insufficiently polymorphic for the method to work well: many co-infections have the same allele as this locus. This supports the use of *P vivax* MS16 for the primary analysis. We also ran the analysis with and without the frequent *P vivax msp1*F3 alleles.

**Table 2:** Genotypes detected in 143 children with at least one blood sample at each routine time-point.

	*Pf msp2*	*Pv* MS16	*Pv msp1*F3
Number of genotypes per child, median (90% central range)	4 (1,11)*	12 (2,23)	1 (0, 9)**
Number of children with each genotype, median (90% central range)	4 (1,36)*	13 (1,48)	1 (0, 36)**

*Omitting the 5 most frequent alleles

** Omitting the 4 most frequent alleles

To describe the observed longitudinal patterns, we summarize data from the 143 children with at least one visit at each of the 9 routine study time-points.

There were 1837 *P vivax* MS16 genotypes detected in 143 children with samples at each routine time-point: of the genotypes detected, 1283 (69%) were observed in one interval per child (e.g. pattern 00010000), 389 (21%) in two intervals (e.g. pattern 01000100), 124 (7%) in three and 41 (2%) in four or more. The maximum number of intervals in which an allele was detected in an individual child was 7. The majority of these infections were detected through the routine surveys only (1256, 68%) or both routine and sick visits (303, 16%) but 278 (15%) genotypes were only seen at the sick visits. Genotypes detected more than once in a child, were detected again most frequently in the intervals immediately following their first detection (Table 3). Otherwise they tended to be present in one interval and not the next, consistent with the high levels of treatment which truncates longer durations, or short durations of patent blood-stage infections.

**Table 3:** Proportion of *P vivax* MS16 genotypes observed in different intervals by the interval first seen*.

					Interval						
		N genotypes	1	2	3	4	5	6	7	8	9
	1	142	100%	22%	11%	12%	7%	4%	9%	5%	5%
interval	2	255		100%	15%	12%	13%	7%	8%	7%	5%
first	3	230			100%	20%	12%	7%	10%	6%	3%
observed	4	227				100%	19%	8%	4%	6%	3%
	5	232					100%	21%	11%	11%	6%
	6	219						100%	16%	11%	6%
	7	244							100%	12%	9%
	8	186								100%	16%
	9	102									100%

*In 143 children with at least one visit at each routine time-point. The first interval includes the first routine time-point only (ie no illness visits).

### Results: Model estimates

#### Simulated data

To validate our method, we simulated data for specified parameter values (S3 Text). The estimation procedure was able to recover the values reasonably well.

#### Ilaita I cohort

**Fig 2:**
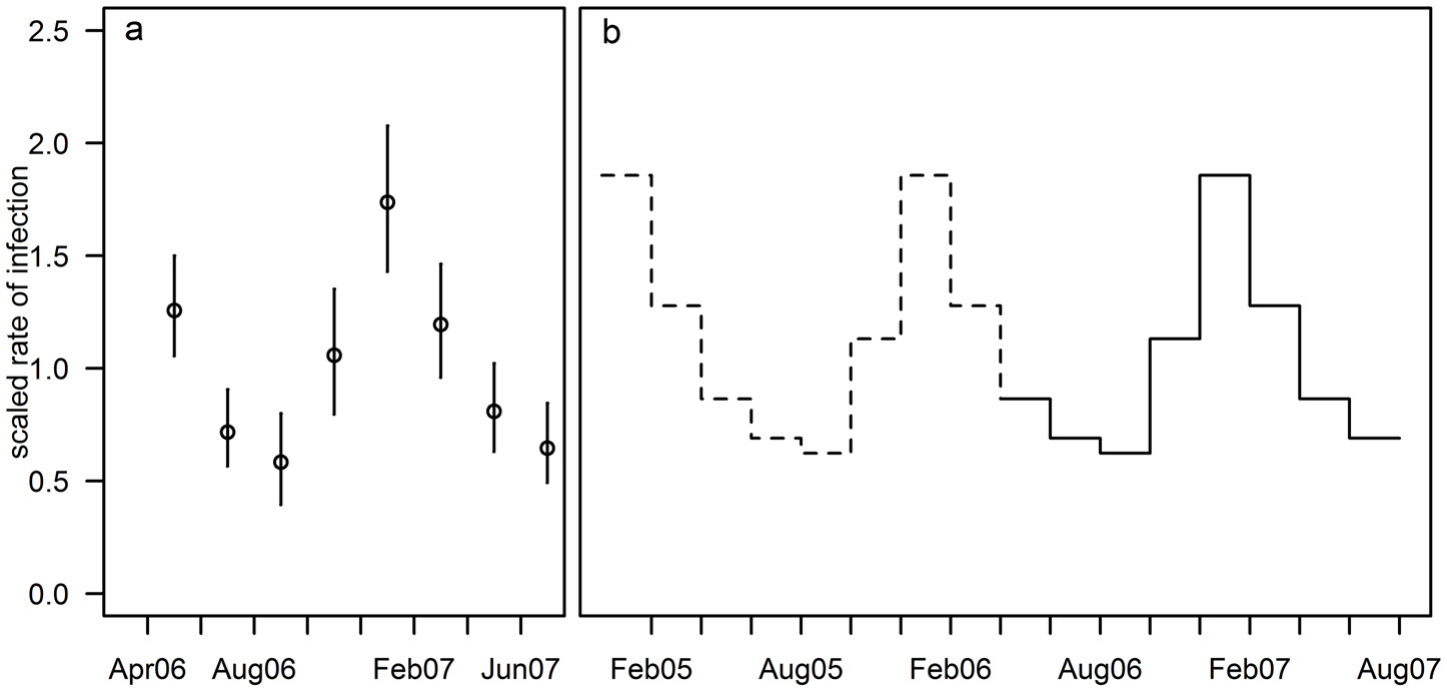
Estimated seasonal pattern of *P falciparum* infections (a) estimated pattern during study follow-up (b) seasonal pattern extended to time preceding the study period. Circles (a): estimated seasonal pattern of *P falciparum* infections (95% CI) during the study period. Solid line (b): inferred seasonal pattern of *P vivax* primary infections during the study period. Dashed line (b): assumed seasonal pattern of *P vivax* primary infections prior to the study period.

The estimated seasonal pattern for the force of infection for *P falciparum* shows a peak in January 2007 (Fig 2a), in agreement with previous estimates by other methods [33]. The estimated seasonal pattern for *P falciparum* was averaged and extended to cover time before the start of the cohort follow-up (Fig 2b).

The estimates for *β*_1_, which multiplies the *P falciparum* seasonality to give the incidence of *P vivax* primary infections, for MS16 suggests that children in this cohort experience a mean of 11.5 (10.5,12.3) primary infections per year (Table 4). The estimates of primary infection were lower for *msp1*F3 than for MS16 reflecting downward biases because superinfections with the same allele do not contribute to the estimate (when very frequent alleles are included) or because a substantial proportion of infections are excluded (when frequent alleles are excluded).

**Table 4:** Parameter estimates and 95% confidence intervals for the *P vivax* model.

	MS16	all *msp1*F3 alleles	*msp1*F3 omitting 4 most common alleles
log likelihood	21431.2	14284.6	7761.7
*β*_1_: Factor for primary infection*	0.015 (.014, 0.016)	0.013 (0.012,0.015)	0.010 (0.008, 0.011)
*Derived from the estimate for β*_1_: the incidence of primary infection/year per for a 3 year old child for all genotypes (as a mean over the seasonal fluctuations over one year):			
	11.5 (10.5,12.3)	5.4 (4.9,6.4)	4.1 (3.1,4.6)
Covariates for the incidence of primary infection			
Incidence rate ratio for bednet use ≥50% compared to <50%**	0.70 (0.63, 0.77)	0.74 (0.66, 0.84)	0.67 (0.56, 0.79)
Incidence rate ratio for Sunuhu compared to Ilaita	1.16 (1.04, 1.28)	1.07 (0.94, 1.20)	1.05 (0.89, 1.23)
*β*_2_: Ratio of incidence of relapses to fixed pattern *f*(*x*)	37.2 (35.5, 39.0)	72.8 (69.6, 76.0)	32.8 (30.3, 35.5)
*Derived from the estimate for β*_2_:			
Incidence of relapse/2 months following an individual primary infection (*β*_2_ *f*(*x*)):			
*γ*_0_	0.27	0.52	0.24
*γ*_1_	1.38	2.70	1.22
*γ*_2_	0.99	1.94	0.88
*γ*_3_	0.61	1.21	0.54
*γ*_4_	0.41	0.80	0.36
*γ*_5_	0.28	0.55	0.25
*γ*_6_	0.20	0.38	0.17
*γ*_7_	0.14	0.27	0.12
*γ*_8_	0.10	0.20	0.09
Mean number of relapses per primary infection***:			
	4.3 (4.0,4.6)	8.3 (8.0,8.8)	3.7 (3.4,4.1)

*The estimates *for P falciparum* excluded the most frequent alleles and were scaled, and so this constant cannot easily be interpreted as a comparison between the incidence of *P vivax* and *P falciparum* infections

** Bednet use was assumed to apply to both the pre-study and study periods

Analysis based on 243 children present at six or more routine time-points

*** Relapses may occur when a blood-stage infection from the same genotype is present or during the prophylactic period and so this is not necessarily the number of distinct blood-stage infections

Bednet use was associated with a decrease in primary infections. Transmission intensity is known to vary with village of residence in this study area, with a smaller village called Sunuhu having a slightly higher transmission intensity compared to Ilaita [13]. This was also apparent in our analysis.

The estimated incidence of relapse by time from primary infection, produced by *β*_2_ and the lognormal distribution, implies that the mean number of relapses per primary infection is estimated to be 4.3 (4.0, 4.6) using MS16. This estimate is consistent with other estimates [4,34], allowing for some relapses to be unobserved if they occur when a blood-stage infection by the same genotype is already present. The estimates from *msp1*F3 for relapses are higher when all alleles are included (probably as a result of re-infection with the same genotype), but similar when the more frequent alleles have been omitted.

Since there were high levels of treatment in the cohort, we were unable to estimate the duration of blood-stage infection. We substituted estimates from data from malaria therapy in non-immune adults in the models to ensure identifiability. A sensitivity analysis indicated that specifying a higher clearance rate would lead to higher estimates for the incidence rates. A higher clearance rate of 2 per two-month interval, equivalent to a mean blood-stage infection duration of 30 days, resulted in an estimated incidence of primary infections of 13.3 (11.9,14.2) per year for a three-year old girl compared to 11.5 (10.5, 12.3) with the clearance rate set at 0.8 from malaria therapy data; the mean number of relapses per primary infection was 5.9 (5.6, 6.2) compared to 4.3 (4.0, 4.6).

Age was accounted for via body surface area for primary infections. Including an effect of age on the incidence of relapse did not substantially change the estimates. The ratio for the incidence of relapses in children 36 months and over compared to under 36 months was estimated to be 1.17 (1.04, 1.30) for MS16, adjusting for relapses within 5 months of the primary infection and 6–16 months lead to a more complicated picture (0.95 (0.82, 1.11) and 1.65 (1.36, 2.00) respectively). These effects are consistent for MS16 and *msp1*F3 and are significant but relatively small and do not substantially alter the overall shape of the distribution. They are also difficult to interpret as, while there could be a true age effect, there could also be compensation for residual confounding with body surface area approximations or assumptions about seasonality.

We attempted to adjust the *P vivax* incidence of relapse for the presence of a *P falciparum* fever, any malaria fever, or any fever, in the previous interval. Unfortunately, the two month intervals were too long relative to the two week time period of the hypothesis [4] and the results were difficult to interpret.

**Fig 3:**
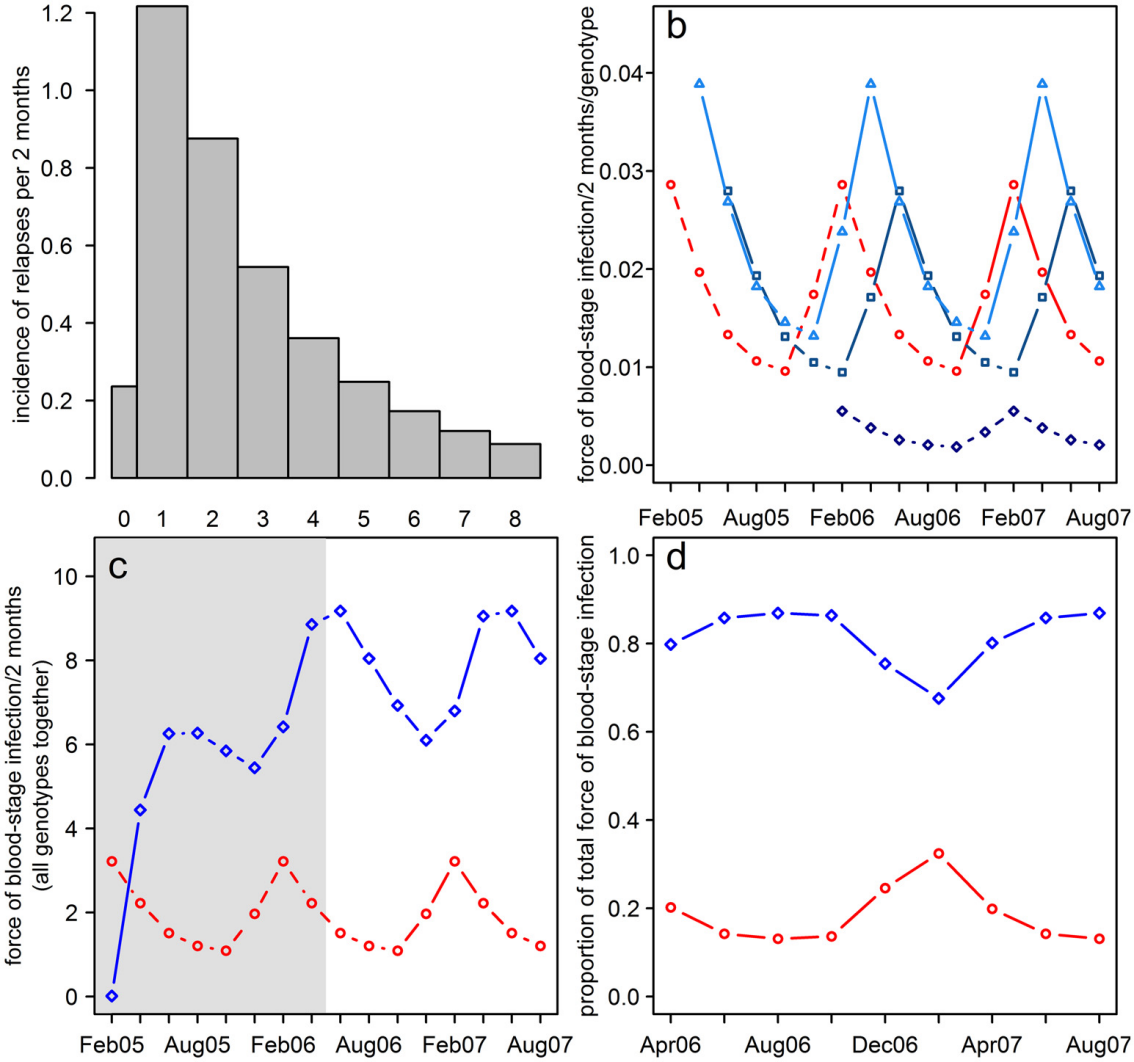
Predicted *P vivax* relapse and primary infection for a three-year-old girl. (a) Estimated incidence of relapse per infection by two month interval from the primary infection. (b) Estimated incidence of primary infection and of relapses subsequent to primary infections one, two and six intervals earlier. Red circles: The incidence of primary infections prior to (dotted line) and during (solid line) the study period. Blue lines: The incidence of relapses subsequent to primary infections occurring one (light blue triangles), two (mid-blue squares) and six (dark blue diamonds) intervals earlier. (c) Estimated force of blood-stage infection from primary infections and relapses. Red circles: primary infections, blue diamonds: relapses. Shaded area is the warm-up period prior to the study, unshaded area is the study period itself. (d) Estimated proportion of the force of blood-stage infection from primary infections and relapses during the study period. Red triangles: primary infections, blue diamonds: relapses. The predictions are for a three-year old girl in Ilaita village with no ITN use using the results for MS16.

The estimated incidence of relapse by interval since primary infection is shown in Fig 3a using the estimates for MS16. The estimated incidence of relapses for a given number of intervals following the primary infections shows lags in the seasonal pattern (Fig 3b). The total incidence of relapse per interval is obtained by summing the relapses falling in each interval (Fig 3c). The pre-study period acts as a warm-up period and our interest lies in the study period itself. Relapses contribute substantially to the force of blood stage infection with the peak incidence of relapse falling in the two month interval after that of primary infection. The proportion of the force of blood-stage infections contributed by primary infections and relapses (Fig 3d) is seasonal, with relapses contributing between 71% and 90% of the total.

Transforming the estimated parameters into the force of blood-stage infection experienced by a three year old child, we estimate the total to be approximately 50 per year at risk using MS16 and *msp1*F3 including all alleles. The molecular force of blood stage infection for *P vivax* in this population has been previously estimated using MS16 as 13.8 (12.8, 14.8) blood-stage infections per child per year-at-risk [14]. Our estimates are substantially higher, and this is expected since we allow for detectability, the possibility of infections being acquired and cleared between the two monthly routine time-points, for multiple relapses per two month interval, and relapses occurring when blood-stage infections by the same genotype are already present. The incidence of distinct blood-stage infections would be substantially lower.

## Discussion

We have estimated the contribution and seasonality of primary infections and relapses by fitting a model of the dynamics of *P vivax* infections to data from a cohort of children in Papua New Guinea.

The seasonality of relapses may have implications for the timing of control strategies, as recently suggested [35]. Mass drug administration with primaquine to clear the hypnozoites would avert the greatest number of relapses if given after the peak transmission season. However if given together with a blood-stage drug with the aim of hitting hardest when onward transmission is lowest, then the dry season may be most appropriate.

These findings aid the interpretation of estimates from two cohort studies conducted in neighbouring regions in Papua New Guinea. In an initial study by Betuela *et al* [36], children aged one to five years who received primaquine showed a 44% reduction in the time to reinfection compared to children who did not receive primaquine. This is substantially less than our model would predict for the same period of the year (approximately 83% for April to July). Together with the very rapid recurrence of *P*. *vivax* infections in the primaquine arm (median 30 days), this suggests the treatment used in that study (artesunate plus primaquine 0.5mg/kg/d for 14 days) had poor efficacy in removing all hypnozoites in these children. Robinson *et al* [37] randomized children between 5 to 10 years of age to a highly efficacious anti-hypnozoite treatment of chloroquine, arthemeter-lumefantrine plus either primaquine (20d, 0.5 mg) or placebo. This trial demonstrated an 83% reduction in risk of recurrent parasitaemia in children that received the primaquine, leading to estimates of the numbers of relapses associated with each primary infection similar to those of the present analyses. The Robinson *et al* cohort were given liver-stage treatment in August. Our analysis suggests that the relative contribution of relapses to the force of blood-stage infection is greatest during this time with a value of 90%. Our results predict that had the study started in February, the proportion contributed by relapses would be 21% lower. Further studies also support a substantial contribution of relapses to blood-stage infection in parasites originating from this region [38,39].

The similarity of the results by Robinson *et al* [37] and the present study lends weight to the findings. The approaches are different but complementary: the primaquine study is a powerful and direct design, but can suffer from drug failure especially in participants with mutations in the CYP2D6 gene [40]. The analysis here can estimate the incidence of primary infections and relapses avoiding the need for primaquine, over longer follow up periods and incorporating seasonality.

We did not observe substantial differences in relapse by age in this cohort. A previous study in the same cohort found that estimates of the force of molecular blood stage infection (molFOB) were similar in different age groups [14]. There is little trend in parasite prevalence across this age range but clinical episodes decreased substantially with age [13]. The rate of relapses may be an inherent characteristic of parasites from Papua New Guinea, and not influenced by host age or immune status. There is a need to investigate relapses in cohorts including older children with greater acquired immunity.

We did not include recombination in the model. A recent whole genome analysis of three consecutive relapses in a traveler infected in Sudan showed that the relapses were meiotic siblings [12]. Recombination between genotypes with the same MS16 or *msp1*F3 allele would not affect the analysis. However recombination between genotypes with different alleles would mean that there would be higher numbers of relapses per infection: the model would overestimate the incidence of primary infection and underestimate the incidence of relapse. If every infection was comprised of two genotypes then there would be roughly twice as many relapses per infection as estimated. Preliminary extensions to the model to take the frequency of recombination into account required excessive computational power.

Systemic fevers, including those of *P falciparum*, have been associated with *P vivax* relapses [4,5,41,42]. We were unable to investigate an association between *P vivax* incidence of relapse for the presence of a *P falciparum* fever, or any malaria fever, or any fever, in the previous interval (results section) with this study design. We also do not take into account potential interactions between *P falciparum* and *P vivax*, or between *P vivax* genotypes: these are the subject of further work.

We approximated the seasonality of *P vivax* primary infections with that of *P falciparum*. This approximation appears to be reasonable for the Wosera in Papua New Guinea, but entomological data from the study site itself or further settings would provide additional support. The cohort also ages over the follow-up period. It is possible that the estimates of seasonality could be confounded by differential acquired immunity to *P falciparum* and *P vivax* pre-erythrocytic immunity. However, this effect has been estimated to be small for *P falciparum* [43]. The estimated seasonality could also be affected by the assumption that detectability is the same all year round: detectability may be greater in the rainy season for the major allele when parasite densities are higher but may be reduced for minority alleles as MOI increases. We assume that the seasonal pattern was the same as during the study period in the years before the study, which ignores variation in seasonality between years. We did not account for uncertainty in the *P falciparum* seasonality when estimating the *P vivax* parameters. We did not attempt simultaneous estimation of *P falciparum* seasonality and the *P vivax* parameters: the computational requirements for the *P vivax* model alone were challenging. However a sensitivity analysis showed that small changes in *P falciparum* seasonality did not alter the conclusions for *P vivax*.

We were unable to estimate the natural clearance rate of untreated infections in this cohort since antimalarial treatment was frequent. High levels of treatment have also been described elsewhere in Papua New Guinea [44]. We substituted estimates from data from malaria therapy in non-immune adults in the models to ensure identifiability. Fixing the value of the natural clearance rate affects the estimates for the incidence of primary infection and relapse. A sensitivity analysis indicated that specifying a higher clearance rate would lead to higher estimates for the incidence rates. Preliminary analyses of other cohorts in Papua New Guinea have suggested a shorter duration than that observed in malaria therapy patients. We also adopted an exponential distribution for simplicity of the model. We recognize that this has been shown to be incorrect for *P falciparum* [6,45] however the frequency of treatment does mean that assumptions about the distribution of the duration of infections are unlikely to be critical.

We based our model on the frequency of genotype patterns observed, following previous models of *P falciparum* [6,8,9]. The development of the model was driven by the biology of *P vivax* and the need to include covariates and account for missing timepoints. The probabilities of the observed patterns must be calculated for each combination of age, ITN use, treatments administered, village and missing timepoints. Since these combinations refer frequently to only one child in the study, the observed probabilities must be calculated for each child. As a consequence, the computational demands are high. However this allows more flexibility and future models could move farther away from patterns based on fixed intervals to continuous time. Despite the limitations, the current model is suitable for the analyses shown here. To our knowledge, this is the first time the contributions have been teased out in an endemic setting without the use of primaquine.

The prevalence of *P vivax* is usually considered to be less seasonal than that of *P falciparum*. In this paper, we have shown that this is composed of the different seasonal patterns of primary infections and relapses. Our analysis supports a substantial contribution of relapses to the force of blood-stage infection in Papua New Guinea underscoring the need for effective intervention strategies against liver-stage parasites.

## Supporting Information

S1 Text The assumption that the seasonality of *P falciparum* infections can represent the seasonality of *P vivax* primary infections. (DOCX)

S2 Text The distribution of *P vivax* relapse times in the Chesson strain from New Guinea. (DOCX)

S3 Text Evaluation of model performance. (DOCX)

S1 Table List of quantities in the model. (DOCX)

S1 Checklist STROBE checklist. (DOC)
