# Supplementary material for: The Incidence and Differential Seasonal Patterns of Plasmodium vivax Primary Infections and Relapses in a Cohort of Children in Papua New Guinea
Source: PLoS Negl Trop Dis. 2016 May 4;10(5):e0004582. doi: 10.1371/journal.pntd.0004582 (PMC4856325; doi:10.1371/journal.pntd.0004582)
Supplement: S1 Text — (DOCX) [file pntd.0004582.s001.docx]

**S1 Text**: The assumption that the seasonality of *P falciparum* infections can represent the seasonality of *P vivax* primary infections

We investigated the assumption that the seasonality of *P falciparum* and *P vivax* primary infections are the similar using data from entomology surveys in the Wosera, Papua New Guinea. Monthly mosquito collections were carried out consistently in seven villages between 1990 and 1992. Each village was divided into 5 to 6 sectors. Within each sector, a random inhabitant was selected each month. Human landing catches were carried out inside and outside the house between sunset and sunrise [1]. Indoor and outdoor resting catches were also performed but not included in the analysis in order to focus on feeding mosquitoes. Sporozoite positivity was determined by an ELISA test for circumsporozoite antigen in the head and thorax [2].

Of the 47345 mosquitoes tested for *P vivax,* 160 (0.3%) were positive. For *P falciparum*, 47355 were tested and 343 (0.7%) were positive. The seasonal patterns for *P falciparum* and *P vivax* are similar (Fig A).

| Fig A. Proportion of mosquitoes tested sporozoite positive by monthly survey |
| --- |
|  |

Blue dotted line: *P falciparum*, Red solid line: *P vivax*

1. Hii J, Smith T, Mai A, Mellor S, Lewis D, Alexander N, et al. Spatial and temporal variation in abundance of Anopheles (Diptera: Culicidae) in a malaria endemic area in Papua New Guinea. J Med Entomol 1997;34:193–205.

2. Hii J, Smith T, Vounatsou P, Alexander N, Mai A, Ibam E, et al. Area effects of bednet use in a malaria-endemic area in Papua New Guinea. Trans Roy Soc Trop Med Hyg 2001;95:7–13.
