# Supplementary material for: The Incidence and Differential Seasonal Patterns of Plasmodium vivax Primary Infections and Relapses in a Cohort of Children in Papua New Guinea
Source: PLoS Negl Trop Dis. 2016 May 4;10(5):e0004582. doi: 10.1371/journal.pntd.0004582 (PMC4856325; doi:10.1371/journal.pntd.0004582)
Supplement: S2 Text — (DOCX) [file pntd.0004582.s002.docx]

**S2 Text. The distribution of *P vivax* relapse times in the Chesson strain from New Guinea**

We estimated the distribution of relapse times using data on deliberately infected volunteers. A total of 207 volunteers from two Federal penal institutions in Atlanta, Georgia and Seagoville, Texas were infected with the Chesson strain of *P vivax* via sporozoites. The Chesson strain came from a soldier infected in New Guinea [1]. In prison volunteers with a single exposure date, 802 attacks were observed up to 18 months of follow-up. Most volunteers were treated promptly and followed up for the full 18 months and most did not have periods of prophylaxis. Blood smears were made daily from the eight to 60^th^ day after exposure with minor exceptions, then two to three times weekly up to 12 months and weekly up to 18 months. Details are given elsewhere [2,3]. The number of relapses per week from the time of inoculation was extracted from the publication [3] (Fig B). We fit a number of distributions and found that the best fit was given by a lognormal distribution with $\mu_{g}$=2.92 weeks in log-units and $\sigma=$0.956. We summed the values within each two month interval and scaled them to give $f(x)$, the pattern of the incidence of relapses. We allowed for 1.5 weeks from inoculation to patent primary infection.

| Fig B. The proportion of the total relapses occurring in each week in US prison volunteers | |  | |
| --- | --- | --- | --- |
|  |  | |  |

Our distribution is supported by a previous study in which a model was fitted to data from Australian soldiers returning from East Timor [4]. Infection dates were not known exactly. The fitted distribution was a four-parameter Weibull, which has a slightly later peak, but an approximately similar shape to the lognormal. Further support for the very early part of this distribution comes from estimates of the time to first relapse [5–7].

1. White N. Determinants of relapse periodicity in *Plasmodium vivax* malaria. Malar J 2011;11:297.

2. Coatney, GR, Cooper, WC. Studies in malaria VI. The organization of a program for testing potential antimalarial drugs in prisoner volunteers. Am J Hyg 1948;47:113–9.

3. Coatney, RG, Cooper, WC, Young, MD. Studies in human malaria XXX. A summary of 204 sporozoite-induced infections with the Chesson strain of *Plasmodium vivax*. J Nat Malar Soc 1950;9:381–96.

4. Chen N, Auliff A, Rieckmann K, Gatton M, Cheng Q. Relapses of *Plasmodium vivax* infection results from clonal hypnozoites activated at predetermined intervals. J Inf Dis 2007;195:934–41.

5. Battle K, Karhunen M, Bhatt S, Gething P, Howes R, Golding N, et al. Geographic variation in *Plasmodium vivax* relapse. Mal J 2014;13:144.

6. Lover A, Coker R. Quantifying the effect of geographic location on epidemiology of *Plasmodium vivax* malaria. Emerg Inf Dis 2013;19:1058–65.

7. Lover A, Zhao X, Gao Z, Coker R, Cook A. The distribution of incubation and relapse times in experimental human infection with the malaria parasite *Plasmodium vivax*. BMC Infect Dis 2014;14:539.
