# Supplementary material for: The Incidence and Differential Seasonal Patterns of Plasmodium vivax Primary Infections and Relapses in a Cohort of Children in Papua New Guinea
Source: PLoS Negl Trop Dis. 2016 May 4;10(5):e0004582. doi: 10.1371/journal.pntd.0004582 (PMC4856325; doi:10.1371/journal.pntd.0004582)
Supplement: S3 Text — (DOCX) [file pntd.0004582.s003.docx]

**S3 Text. Evaluation of model performance**

1. **How well can the model recover parameter values from simulated data?**

We simulated data to test how well the model can recover parameter values. We simulated a dataset of 100000 child-genotype observations. The estimated seasonality for *P falciparum* from the Ilaita cohort was used. The simulated children were 3 years old at enrolment and body surface area was accounted for but other covariates including treatment were not included. There was no reinfection with the same genotype. The incidence of relapse by time from primary infection was allowed to vary freely. We simulated one blood sample per routine time-point with no missing observations. We set the rate of clearance to be 0.8 per interval with no treatment, and detectability, $q$, to be 0.8, which is similar to the probability of detection in the cohort for a time-point with two blood samples.

Table A. Recovered parameter estimates for simulated data

|  | True values | Estimated values |
| --- | --- | --- |
| $\beta_{1}$ | 0.05 | 0.05 |
| $\gamma_{0}$ | 0 | 0.048 |
| $\gamma_{1}$ | 0.1 | 0.07 |
| $\gamma_{2}$ | 0.2 | 0.22 |
| $\gamma_{3}$ | 0.3 | 0.29 |
| $\gamma_{4}$ | 0.6 | 0.62 |
| $\gamma_{5}$ | 0.3 | 0.34 |
| $\gamma_{6}$ | 0.1 | 0.07 |
| $\gamma_{7}$ | 0.05 | 0.056 |
| $\gamma_{8}$ | 0.02 | 0.007 |

The model was able to reproduce the true values reasonably well.

1. **How well does the model fit the cohort data?**

We plot the predicted probability against the observed frequencies of patterns for analysis of the *P vivax* marker MS16. The largest number of observed patterns, and the largest predicted probability, correspond to the zero pattern (Fig Ca). Omitting this point, the fit to the other patterns also appears to be good with a positive correlation between the predicted pattern and the frequency of observed patterns (Fig Cb).

Fig C. Predicted probabilities against observed proportion of patterns for MS16

1. All patterns (b) Excluding the zero pattern, with a square-root scale for visibility.

The predicted probabilities were calculated separately for each child, to take their treatment, age and number of visits at each routine time-point into account, and then summed. 143 children with at least one visit at each routine time-point were included.
