# Supplementary material for: The Incidence and Differential Seasonal Patterns of Plasmodium vivax Primary Infections and Relapses in a Cohort of Children in Papua New Guinea
Source: PLoS Negl Trop Dis. 2016 May 4;10(5):e0004582. doi: 10.1371/journal.pntd.0004582 (PMC4856325; doi:10.1371/journal.pntd.0004582)
Supplement: S1 Table — (DOCX) [file pntd.0004582.s004.docx]

S1 Table. Quantities used in the model

|  | Description | Source of information |
| --- | --- | --- |
| ***General quantities*** | |  |
| $C$ | Number of children | 1 |
| $t_{cs}$ | True pattern $s$ in child $c$ | 3 |
| $d_{csi}$ | True state at time-point $i$, pattern $s$, child $c$ | 3 |
| $o_{cw}$ | Observed pattern $w$ in child $c$ | 1 |
| $b_{cwi}$ | Observed state at time-point $i$ of pattern $w$ in child $c$ | 1 |
| $n_{cw}$ | Frequency of pattern $w$ in child $c$ | 1 |
| $q_{g}$ | Probability of detecting a genotype in a single blood sample positive by microscopy or LDR, given that it is present | 4 |
| ${1-q}_{k}$ | Probability of not detecting a genotype in a single blood sample negative by microscopy or LDR | 4 |
| $g_{ci}$ | Number of blood samples at a routine time-point positive by microscopy or LDR and genotyped | 1 |
| $k_{ci}$ | Number of blood samples at a routine time-point negative by microscopy or LDR and not genotyped | 1 |
| $z_{ci}$ | Probability of detecting a genotype at routine time-point $i$, given that it is present | 4 |
| $m_{ci}$ | Duration at risk for interval $i$ for child $c$ in 2 month intervals | 1 |
|  |  |  |
| ***Quantities relating to P falciparum model*** | |  |
| $\lambda_{fi}$ | Incidence of *P falciparum* infection in interval *i* (input to *P vivax* model) | 2 |
| $\lambda_{fci}$ | Incidence of *P falciparum* infection in interval $i$ for child $c$ | 3 |
| $\mu_{f}$ | Rate of clearance of *P falciparum* infection in 2 month intervals | 3 |
| $p_{fci}^{10},p_{fci}^{11},p_{fci}^{00} ,p_{fci}^{01}$ | Probabilities of transitions for *P falciparum* for interval $i$ for child $c$ | 3 |
|  |  |  |
| ***Quantities relating to P vivax model*** | |  |
| $p_{yc}$ | Probability per genotype that primary infection occurs in interval $y$ in child $c$ | 3 |
| $\beta_{1}$ | Ratio of $\lambda_{vi}$ to scaled $\lambda_{fi}$ | 2 |
| $\lambda_{vci}$ | Incidence of *P vivax* primary infection per genotype in interval $i$ for child $c$ | 3 |
| $\mu_{b}$ | Clearance rate of blood-stage *P vivax* genotype in 2 month intervals | 4 |
| $\gamma_{yci}$ | Incidence of relapse in interval $i$ following primary infection in interval $y$ per genotype for child $c$ | 3 |
| $\beta_{2}$ | Ratio of fitted distribution of relapses from literature to give $\gamma_{iyc}$ | 2 |
| $p_{yci}^{10},p_{yci}^{11},p_{yci}^{00} ,p_{yci}^{01}$ | Probabilities of transitions per genotype for *P vivax*, given that primary infection has occurred | 3 |

1=data, 2=parameter value of interest estimated by the model, 3=intermediate parameters or probability of intermediate parameters estimated in the model, 4=fixed input value*

* The values of the fixed parameters can vary between markers and are described in the methods section
